# Quantification of pedogenic particles masked by geogenic magnetic fraction

**DOI:** 10.1038/s41598-021-94039-1

**Published:** 2021-07-20

**Authors:** Marcin Szuszkiewicz, Hana Grison, Eduard Petrovský, Maria Magdalena Szuszkiewicz, Beata Gołuchowska, Adam Łukasik

**Affiliations:** 1 Institute of Environmental Engineering, Polish Academy of Sciences, 34 M. Skłodowskiej-Curie St, 41-819 Zabrze, Poland; 2 Institute of Geophysics, Czech Academy of Sciences, Boční II/1401, 141 00 Prague 4, Czech Republic; 3 Institute of Environmental Engineering and Biotechnology, Faculty of Natural Sciences and Technology, University of Opole, 6 Kardynała B. Kominka St, 45-032 Opole, Poland

**Keywords:** Geomagnetism, Geophysics

## Abstract

Pedogenic magnetic fraction in soils is attributed to fine-grained particles, i.e. superparamagnetic grains. In the case of a strongly magnetic geogenic fraction, pedogenic magnetic contribution is hard to detect. To the best of our knowledge, detailed research into the masking of pedogenic superparamagnetic grains and quantification of this effect has not yet been carried out. The principal aim of our research is to quantify the influence of coarse-grained ferrimagnetic fraction on the detection of the superparamagnetic grains. In order to describe the masking phenomenon, volume and frequency-dependent magnetic susceptibility were determined on a set of laboratory prepared samples composed of natural substances: a diamagnetic quartz matrix, detrital coarse-grained ferrimagnetic crystals from alkaline and ultra-alkaline igneous rocks, and superparamagnetic soil concretions formed in the *Haplic Cambisol.* Mineralogy, concentration, type and grain size of the tested material were described by parameters of environmental magnetism. The magnetic parameters distinguish both geogenic multidomain and pedogenic superparamagnetic grains. The magnetic signal of the superparamagnetic grains is gradually masked by the increasing proportion of multidomain grains of magnetite/maghemite. The experiment clearly describes the masking effect and brings new insight to studies dealing with strongly magnetic soils of natural and/or highly contaminated origin as a tool for estimation of superparamagnetic pedogenic contribution.

## Introduction

Occurrence of naturally formed ferromagnetic minerals sensu lato (i.e. ferrimagnetic and canted antiferromagnetic) as a result of pedogenesis may affect the vertical distribution of the magnetic susceptibility values in soils. Most research has focused on ferrimagnetic phases (the so-called ‘soft magnetic fraction’) such as magnetite (Fe_3_O_4_) or maghemite (γ-Fe_2_O_3_), which have been identified as the main carriers of the soil magnetic susceptibility enhancement^1–7^. However, antiferromagnetic iron oxide minerals (the so-called ‘hard magnetic fraction’) like haematite (α-Fe_2_O_3_), goethite (α-FeOOH), lepidocrocite (γ-FeOOH) or ferrihydrite (Fe_5_HO_8_·4H_2_O) are generally much more abundant in soils than ferrimagnetic minerals^8^.

Iron oxides play an essential role in pedogenic processes, supplying information about distribution, migration, and transformation of iron minerals in soils^9^. The type of iron oxide minerals, which will be formed, depends on the iron ions’ oxidation state, i.e. the ratio of the Fe^2+^ ions to the Fe^3+^, related to such soil parameters as oxidation rate, the content of organic acids and pH, as well as the process of hydrolysis^8^. Pedogenic iron oxide minerals in soils are attributed to nanoparticles and the processes of their formation fall within the group of medium-rate development from 10^3^ to 10^4^ years^10^. The pedogenic magnetite can be formed both in inorganic and biogenic processes, e.g. from ferrihydrite in the microbially mediated Fe^3+^ reduction^11^ or during its intracellular production by magnetotactic bacteria^12^.

It is a well-known fact that the magnetic susceptibility of the soil is related to the specific factors ranked from the most important to the least important: mineral concentration ≫ mineral composition > crystal size > the shape of ferrimagnetic phases of both natural (geogenic and pedogenic) and artificial (e.g. technogenic) origin^13^. The distinction between geo-, pedo-, and technogenic magnetic signal works well in soils developed from non-magnetic or weakly magnetic bedrocks, but in the case of strong geogenic contribution can still be problematic^14–21^. The same applies to the coexistence of different sources of magnetic particles, when the domination of the magnetic signal of secondary over primary minerals may occur^22–24^. The secondary magnetic minerals in the soil, including the secondary ferrimagnetic minerals (SFMs), can be pedo- and biogenic^22^, or anthropogenic in origin as technogenic magnetic particles (TMPs^25,26^). By contrast, the primary magnetic minerals are represented by geogenic magnetic particles (GMPs^20^). The frequency-dependent magnetic susceptibility of the pedogenic magnetic fraction^27–29^ can be efficiently suppressed (masked) by the presence of frequency-independent and/or weakly frequency-dependent ferrimagnetic grains, or a significant amount of (frequency-independent) paramagnetic components, which account for over 50% of the total magnetic susceptibility signal^13^. On the other hand, the reason for this phenomenon could be the pedogenic components consisting of a mixture of superparamagnetic (SP), single-domain (SD)^30^ and pseudo-single-domain (PSD) grains^31^.

To the best of our knowledge, there has been no detailed research into the masking phenomenon of pedogenic superparamagnetic grains and quantification of this effect so far. Up to now, the presented models of SP/stable SD magnetic enhancement^11,32–34^ cannot be applied to estimate the absolute amount of pedogenic magnetic fraction or to explain the mechanism of the masking phenomenon. Nonetheless, a mixing experiment suggests that the frequency-dependent magnetic susceptibility may be used semi-quantitatively to estimate the proportion of SP grains in a sample^22^.

The principal aim of this research was to show the influence of the coarse-grained ferrimagnetic fraction on the detection of the very fine superparamagnetic grains. For this purpose, the frequency-dependent magnetic susceptibility was used, i.e. the parameter which reflects the presence of the grains near the SP/stable SD boundary (~ 20 nm in diameter). Consequently, to understand and describe the phenomenon of the ‘masking effect,’ research into its modelling was initiated.

## Materials and methods

### Environmental settings and sampling

#### Quartz (Q)

Milky quartz, the most common variety of quartz, i.e. a crystal form of silicon dioxide (SiO_2_), was collected in the abandoned vein quartz mine ‘Stanisław’ located in Szklarska Poręba on the south slope and a ridge of the Izerskie Garby Mountain (the central-east part of the Izera Mountains, southwestern Poland; see Supplementary Table S1). The milky quartz crystals were up to 10 cm in length.

#### Magnetite/maghemite (M)

A crystal form of primary iron oxide (Fe_3_O_4_/γ-Fe_2_O_3_) was collected from Quaternary deposits (i.e. the horizon of opaque heavy minerals) of the Baltic Sea coast of Poland^35^ (see Supplementary Table S1). Detrital magnetite/maghemite crystals have been found in seashore sand, deriving from alkaline and ultra-alkaline Scandinavian igneous rocks^36^. The horizon of opaque sands of heavy minerals (including magnetite/maghemite) is quite common in this area, making the extraction of natural (geogenic) ferrimagnetic grains easier and cheaper than using lab-made samples.

#### Soil concretions (SCs)

Soil concretions were collected from *Haplic Cambisol* developed from Carboniferous sandstones in the ‘Murckowski’ Forest in Katowice, southern Poland^37^ (see Supplementary Table S1). These spherical soil concretions, looking like ‘pepper grains,’ were formed as a result of residual accumulation in the *cambic* horizon (*B*_*w*_) at depths of 20–30 cm. The concretions were isolated from soil pits and/or soil cores in the under-crown areas of Norway spruce (*Picea abies* Karst.) biogroups. The *B*_*w*_ horizon is rich in soil concretions on the study site, which favors the acquisition of natural (pedogenic) samples with a relatively high concentration of SP grains, with a relatively low content of multidomain (MD) grains.

### Experimental procedures

The composition of the experimental samples, i.e. the contribution of matrix (milky quartz) and components (magnetite/maghemite and/or soil concretions), prepared for the measurements of magnetic susceptibility are presented in Table S2 (see Supplementary).

Milky quartz crystals were crushed and ground in an agate mortar, then were weighed and placed into 10 ml diamagnetic cylindrical containers for magnetic susceptibility measurements. The magnetite/maghemite samples from seashore sand (i.e. opaque heavy minerals horizon) were air-dried (at room temperature) and sieved through a 0.2 mm mesh to separate the coarse sand grains, organic matter, or shell fragments, etc. Next, a one-stage magnetic separation process was carried out using a weak ferrite magnet to isolate ferrimagnetic grains of magnetite/maghemite (up to 0.2 mm in diameter) from antiferro-, para-, and diamagnetic grains (see Supplementary Fig. S1). High clay content in the field sediments may cause problem during separation of magnetic particles^38^, due to the clay particles which stick to the ferrimagnetic grains and may carry the other magnetic phases. In the case of sedimentation processes, the opposite situation is observed. Clay particles are covered by ferrimagnetic nanoparticles and affect the magnetic behavior^39^. Therefore, to reduce these effects after the magnetic separation process, we carefully washed the magnetite/maghemite grains in an ultrasonic bath. The magnetite/maghemite grains were used to prepare 15 samples of increasing low-field volume magnetic susceptibility from 10 to over 500 × 10^–5^; coded M_n_ where M—magnetite/maghemite, n—the approximate value of κ_465Hz_ in ascending order, i.e. M_10_, M_20_, M_25_, M_50_, M_75_, M_100_, M_125_, M_150_, M_175_, M_200_, M_250_, M_300_, M_400_, M_500_, M_> 500_ (see Supplementary Table S2). The M_n_-specimens were created by adding more magnetite/maghemite grains until a specific value of magnetic susceptibility was obtained in a given sample. The collected soil concretions (up to 5 mm in diameter) were air-dried, cleaned using a small brush of soil grains, rock crumbs or root fragments. Every clean concretion was wrapped in foil and placed into a 10 ml container filled with milky quartz for preliminary magnetic susceptibility measurements. Next, the concretions with the highest percentage frequency-dependent magnetic susceptibility values were crushed and ground in an agate mortar. The magnetite/maghemite crystals (15 samples) and ground soil concretions (3 samples) were placed inside diamagnetic, transparent gelatin capsules and immobilized in cotton wool. Afterwards, the capsules in various configurations were placed in 10 ml containers filled with milky quartz for magnetic susceptibility measurements (see Supplementary Table S2). The same material (i.e. SC1, SC2, SC3, and M capsules) was used for further studies that included the hysteresis loops and back-field remagnetization measurements as well as the thermomagnetic analyses.

### Magnetic measurements

In order to test the magnetic characteristic of geogenic and pedogenic samples, the following magnetic parameters were determined: volume magnetic susceptibility (κ_465Hz_ and κ_4650Hz_) and frequency-dependent magnetic susceptibility (κ_fd_ and κ_fd_%), high-temperature dependence of magnetic susceptibility, saturation induced magnetization (Ms), remanent saturation magnetization (Mrs or SIRM), coercive force (Bc) and coercivity of remanence (Bcr).

The magnetic mineralogy of the investigated samples was determined by the Curie point (Curie temperature—T_C_) estimation from thermomagnetic curves (heating and cooling). The Curie point was estimated using the test for paramagnetic Curie–Weiss law at temperatures above T_C_^40,41^. The analyses of the temperature-dependence of magnetic susceptibility of the samples were performed in the ambient air at a heating rate of 8.6 °C/min, and in the temperature range from ~ 40 to ~ 700 °C. Thermomagnetic measurements were carried out using AGICO Kappabridge MFK1-FA equipped with a high temperature furnace CS4 (AGICO Kappabridge MFK1-FA; Advanced Geoscience Instruments Company, Brno, Czech Republic).

The contribution of magnetic minerals in the samples was estimated on the basis of concentration-dependent parameters (κ_465Hz_, SIRM, and Ms), which increase monotonically with the amount of magnetic phase present in a monomineral sample^42^. Moreover, the κ is approximately proportional to the contribution of ferrimagnetic phases in a sample, while, the SIRM is a parameter of the volume concentration of magnetic phases in a sample, but it is also sensitive to grain size changes^27^. In turn, the parameters κ_fd_ and κ_fd_% were used to assess the relative significance of ultrafine nanosized superparamagnetic grains. Whereas, the Bcr/Bc and Mrs/Ms ratios express differences in magnetic mineralogy and grain size^43^. A high value of the Mrs/Ms ratio reflects the dominance of non-interacting SD grains^44^, while, the Bcr/Bc and Mrs/Ms ratios express differences in magnetic mineralogy and grain size^43^.

Measurements of low-field κ were performed using a MS2 Bartington meter equipped with a MS2B dual frequency (low = 465 Hz and high = 4650 Hz) sensor on the 0.1 scale (Bartington Instruments Ltd., Witney, Oxon, UK), which is the most often used instrument for the frequency-dependent magnetic susceptibility measurements despite its limitations (e.g. sensitivity, temperature drift etc.^13^). The frequency-dependent magnetic susceptibility is either expressed as an absolute change of magnetic susceptibility (κ_fd_) Eq. (1)^13^, or a percentage loss of magnetic susceptibility (κ_fd_%) Eq. (2)^13^:

1 $$\kappa_{fd} = \kappa_{465Hz} - \kappa_{4650Hz} ,\;{\text{expressed}}\;{\text{in}}\;10^{ - 5} \;{\text{SI}}$$

2 $$\kappa_{fd} \% = \left( {\frac{{\kappa_{465Hz} - \kappa_{4650Hz} }}{{\kappa_{465Hz} }}} \right),\;{\text{expressed}}\;{\text{in}}\;\%$$

The frequency-dependent magnetic susceptibility estimation for the milky quartz sample as well as specimens 1 and 2 (i.e. M_10_ and M_20_; see Supplementary Table S2) was not carried out due to the sensibility of the instrument^33^. Moreover, due to the samples (i.e. magnetite/maghemite and soil concretion materials) were non-randomly distributed within diamagnetic matrix and may show anisotropy with magnetic susceptibility values partly depending on sample orientation^33^, the samples were measured in the same orientation at both frequencies.

The hysteresis loops were measured (at room temperature) using EV9 Vibrating Sample Magnetometer (DSM Magnetics; ADE Corporation, Lowell, MA, USA) with the maximum applied field of ± 2 T (see Supplementary Fig. S2). The Ms, Mrs, and Bc parameters were obtained by subtracting the linear part of closed branches from the hysteresis loops. However, the Bcr was obtained from the direct current (DC) back-field remagnetization curve in order to remove the remanent saturation magnetization.

The number of magnetic components was estimated according to a method of decomposition of the isothermal remanent magnetization (IRM) acquisition curves and with application of a program which is designed to decompose IRM acquisition curves^45^. The number of magnetic components in this method is evaluated statistically. The combined analysis of the linear acquisition plot (LAP), gradient of the acquisition plot (GAP) and, and the standardized acquisition plot (SAP) versus the logarithm of the applied field provides information on relative contributions of magnetic components in the sample (see Supplementary Figs. S3-S6 and Table S3).

## Results and discussion

### Magnetic response of experimental samples

On the basis of a laboratory-prepared set of samples containing diamagnetic quartz matrix, MD grains of magnetite/maghemite, and SP grains of magnetite/maghemite, experimental magnetic data on κ_465Hz_ and κ_4650Hz_ as well as κ_fd_% and κ_fd_ were obtained (see Supplementary Table S2).

Placing the isolated material in capsules, rather than dispersion in a diamagnetic matrix, may result in magnetostatic interactions. Dispersed powders of SD particles, in which agglomeration of particles occur, show type I behavior, whereas, SD particles in a diamagnetic matrix, in which minimal agglomeration occurs, show type II behavior^46^. Magnetic properties of these two types are significantly different^47^. In samples consisting of magnetite dispersed in a diamagnetic matrix, the interactions are mostly due to particle agglomerations. However, the observed negative character of interactions, yielding a net demagnetizing effect, can be attributed to the specific arrangement of magnetic particles in the specimen M and to a significant contribution of superparamagnetic particles^48^ as in the specimens SC1, SC2, and SC3.

**Figure 1:**
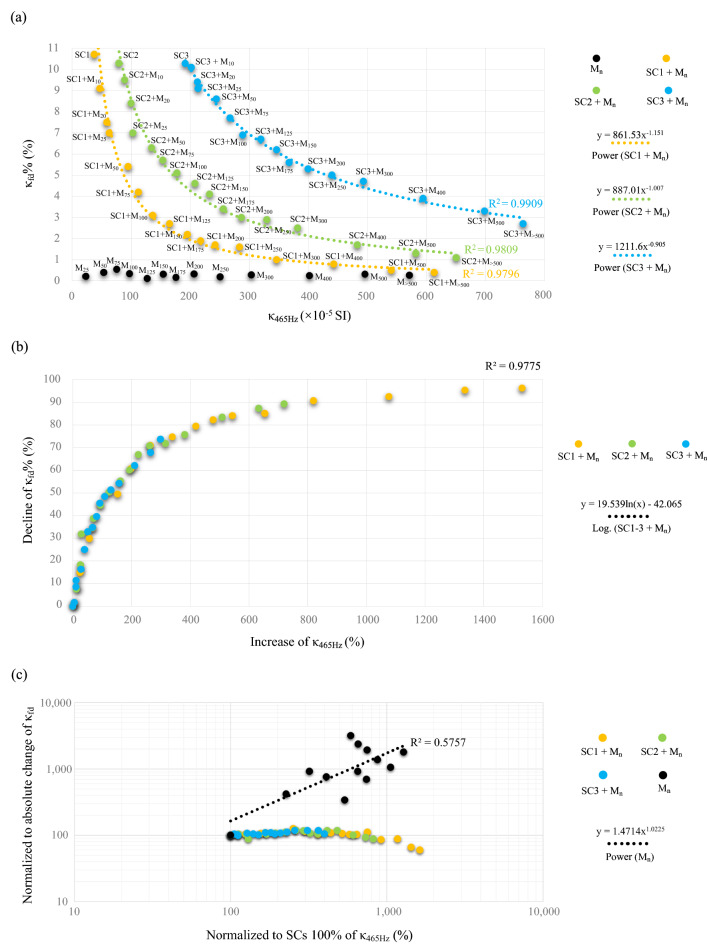
Plots for the experimental data on magnetite/maghemite (M_n_) and soil concretion (SC1-3) samples: (**a**) volume magnetic susceptibility (κ_465Hz_) vs. percentage frequency-dependent magnetic susceptibility (κ_fd_%); (**b**) percentage increase in κ_465Hz_ vs. percentage decline in κ_fd_%; (**c**) bi-logarithmic plot of normalized to SCs of κ_465Hz_ vs. normalized to absolute change of frequency-dependent magnetic susceptibility (κ_fd_).

The magnetite/maghemite samples (specimens 1–15; see Supplementary Table S2) show significantly scattered κ_465Hz_ values (from 9.4 to 571.5 × 10^–5^ SI) and relatively low and constant values of κ_fd_% (0.11–0.71%). In contrast, samples containing mixtures of magnetite/maghemite (M_n_) and soil concretions (specimens: 17–31 for SC1, 33–47 for SC2, 49–63 for SC3; see Supplementary Table S2) are characterized by the values of κ_465Hz_ from 47.0 to 763.8 × 10^–5^ SI, and κ_fd_% values varying from 0.4 to 10.7%. The wide range of κ_465Hz_ and κ_fd_% values is related to the gradual increase in the contribution of MD grains in the specimens. As the amount of MD grains increases, the κ_465Hz_ rises and κ_fd_% drops (Fig. 1a and see Supplementary Table S2). Our experimental results are in agreement with the observations of other authors that samples with κ_fd_% smaller than 3% are dominated by frequency-independent MD grains; a κ_fd_% between 3 and 5% reflects the presence of in principle frequency-independent and frequency-dependent grains, and the κ_fd_% value equal or greater than 5% indicates that SP grains are present in significant amounts^13,27,34,49^.

The relationship between κ_465Hz_ and κ_fd_% for the experimental data is shown in Fig. 1a. Due to the drop in the values of κ_fd_% with the increase in the values of κ_465Hz_ at a certain rate, we used a power trendline to fit the obtained data and to quantify the masking effect. The trendlines of soil concretion samples (SC1-3) show R^2^ in the range 0.9796–0.9909, which can be considered as a very strong correlation (Fig. 1a). In addition, we calculated the percentage decline in κ_fd_% depending on the percentage increase in κ_465Hz_ for the data set, which is displayed in Fig. 1b. The κ_fd_% values increase rapidly and then stabilize. This trend is best fitted by a logarithmic trendline (yielding R^2^ = 0.9775), indicating a very strong correlation between the two parameters (Fig. 1b).

The percentage frequency-dependent magnetic susceptibility gradually decreases with magnetite/maghemite (M) content in the sample. Based on the experimental data (Fig. 1a,b and see Supplementary Table S2), we estimated how much the initial κ_465Hz_ value should increase in order to reduce significantly the initial κ_fd_% value. In the case of samples containing the SC1 soil concretion (specimens 16–31; see Supplementary Table S2), an increase in the initial value of κ_465Hz_ of approximately 1.2, 1.7, 3.0, and 4.4 times is required to reduce the initial value of κ_fd_% of approximately 1.2, 1.5, 2.5, and 4.0 times, respectively. For SC2 samples (specimens 32–47; see Supplementary Table S2), a decline in the initial value of κ_fd_% of approximately 1.2, 1.6, 2.5, and 4.0 times is associated with an increase in the initial value of κ_465_ of approximately 1.3, 1.7, 2.9, and 4.2 times, respectively. Finally, in the case of SC3 samples (specimens 48–63; see Supplementary Table S2), the initial value of κ_465Hz_ should be increased by approximately 1.3, 1.7, 3.1, and 4.0 times to reduce the initial κ_fd_% value of approximately 1.2, 1.5, 2.6, and 3.8 times, respectively. The experimental measurements were carried out until the κ_fd_% values were below 3% (Fig. 1a and see Supplementary Table S2), indicating a minute influence of SP grains on soil magnetic properties^49^.

In addition, based on the experimental data (see Supplementary Table S2), the bi-logarithmic plot of the distribution normalized to SCs κ_465Hz_ versus normalized to the absolute change in κ_fd_ is shown in Fig. 1c. The absolute change in κ_fd_ of SCs from the initial stage is relatively constant and does not exceed 20% (Fig. 1c). The exception is the sample with the lowest value of κ_465Hz_, SC1, where a noticeable decrease in the absolute change in κ_fd_ is observed, when the contribution of M is very large (i.e. more than 20 times; see Supplementary Table S2). In contrast, the M samples show an increase between κ_465Hz_ and the absolute change (yielding R^2^ = 0.5765; Fig. 1c). However, the scattered absolute change of κ_fd_ values (from 0.05 to 1.57 × 10^–5^ SI; see Supplementary Table S2) as well as R^2^ value are not important. Strictly speaking, such low κ_fd_ values suggest virtually no or less than 10% SP grains in the samples^13^.

The obtained data are important for detecting the contribution of the pedogenic magnetic fraction (i.e. SP grains) in soils derived from strongly magnetic parent material and/or in soils significantly affected by TMPs.

### Magnetic characteristic of environmental samples used for the experiment

#### Magnetic mineralogy of environmental samples

**Figure 2:**
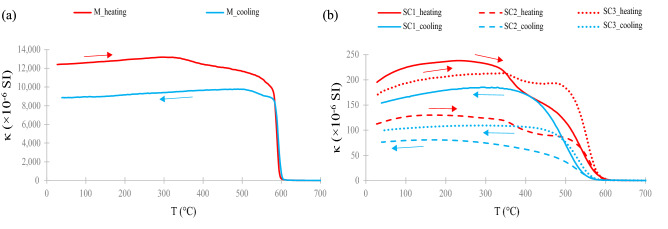
Thermomagnetic measurements of the tested samples: (**a**) magnetite/maghemite (M); (**b**) soil concretions (SC1-3). *T* temperature; *κ* volume magnetic susceptibility.

The curves (heating and cooling) of the temperature dependence of magnetic susceptibility are shown in Fig. 2. Thermomagnetic analysis of the sample M indicates the presence of magnetically monomineral sample with sharp decrease around the Curie point of below 600 °C which suggests narrow grain-size distribution and good crystallinity of the magnetite/maghemite phase (Fig. 2a). Moreover, the sharp decrease around the Curie point and the absence of the haematite-related peak (i.e. very low and constant values of κ above T_C_) for the sample M indicate that the sample represents magnetite/maghemite^50^. The three SC concretion samples (SC1, SC2, and SC3) show in principle similar features as the sample M, but expressed with different intensity. The SC samples show gradual decrease before and around the Curie temperature between ~ 550 and ~ 570 °C, (Fig. 2b). In addition, the thermomagnetic analyses (Fig. 2a,b) show an irreversible behavior in the temperature range below T_C_, and the cooling curves run below the heating curves, which imply that changes occur during heating, i.e. transformation of a strongly magnetic mineral into a mineral with weaker magnetic properties.

#### Magnetic concentration and components in environmental samples

The magnetite/maghemite (M) sample shows a very high κ_465Hz_ value (3221 × 10^–5^ SI), coupled with a very low κ_fd_% value (0.2%) (Table 1). Such a low value of κ_fd_% results from the dominance of non-SP grains (< 10% of the SP contribution)^13^, i.e. coarse MD grains (≥ 30 nm) or extremely fine SP grains (< 5 nm). In contrast, samples of soil concretions (SC1-3) are characterized by much lower values of κ_465Hz_ from 37.7 to 191.6 × 10^–5^ SI, and higher κ_fd_% values ranging from 10.3 to 10.7% (Table 1) suggesting more than 75% of SP grains in the samples^13^. Moreover, pedogenic magnetite may vary in size (from a few to several hundred of nanometers)^11^. Particles below 30 nm will be SP^33^, i.e. they will be able to produce high κ and Ms, but will not retain SIRM and Bc^51^. In contrast, the particles between 30 and 100 nm will be SD or PSD grains. However, the frequency-dependent magnetic susceptibility measured at two frequencies (465 and 4650 Hz) is sensitive only to the presence of magnetite grains between 18 and 20 nm, and not to grains up to 30 nm^51,52^.

**Table 1:** Magnetic parameters (κ_465Hz_—volume magnetic susceptibility; κ_fd_%—percentage frequency-dependent magnetic susceptibility; SIRM (Mrs)—remanent saturation magnetization; Ms—saturation induced magnetization; Bcr—coercivity of remanence; Bc—coercive force) of the tested samples (Q—quartz; M—magnetite/maghemite; SC1-3—soil concretions).

Sample	Formula	Magnetism	κ_465Hz_	κ_fd_%	SIRM (Mrs)	Ms	Bcr	Bc
			(× 10^–5^ SI)	(%)	(mAm^2^ kg^−1^)		(mT)	
Q	SiO_2_	Dia-	− 0.1	–	–	–	–	–
M	Fe_3_O_4_/γ-Fe_2_O_3_	Ferri-	3221.5	0.2	1724.0	55,480.0	36.3	4.6
SC1	Fe_3_O_4_/γ-Fe_2_O_3_	Superpara-	37.7	10.7	100.6	803.6	11.4	3.2
SC2			79.4	10.3	106.0	692.8	16.8	5.4
SC3			191.6	10.3	170.5	963.2	17.5	6.6

We modelled the IRM acquisition curves^45^ for the M and SC1-3 samples (see Supplementary Figs. S3-S6). The predominant mineral phase shows relatively low coercivity component (B_1/2_ = ∼17–56 mT), this phase makes from ∼52 to ~ 76 per cent of the total IRM (see Supplementary Table S3), and is present in M and SC samples. A medium coercivity component (B_1/2_ =  ~ 126–200 mT), constituting from ~ 13 to ~ 48 per cent of the total IRM (see Supplementary Table S3), is also present in all samples. In addition, SC samples contained a high coercivity component (B_1/2_ =  ~ 1000–1122 mT), constituting from ~ 11 to ~ 15 per cent of the total IRM (see Supplementary Table S3). Based on the coercivity distribution^53–56^, we interpreted these three coercivity ranges (from low to high coercivity) as magnetite/maghemite, haematite and goethite, respectively.

The values of the parameters κ_465Hz_, SIRM, and Ms for the soil concretion samples show a similar behavior, which means the highest values of κ_465Hz_ are accompanied by the highest values of SIRM and Ms. There is, however, one exception, the SC1 sample, with the lowest values of κ_465Hz_ and SIRM (37.7 × 10^–5^ SI and 100.6 mAm^2^ kg^−1^), but a moderate Ms value (803.6 mAm^2^ kg^−1^) (Table 1). This may result from the presence of various kinds of magnetic behavior, i.e. different magnetic phases, but with an unquestionable predominance of ferrimagnetics (magnetite/maghemite) in the tested samples (Fig. 2b).

#### Magnetic grain-size analyses in environmental samples

**Figure 3:**
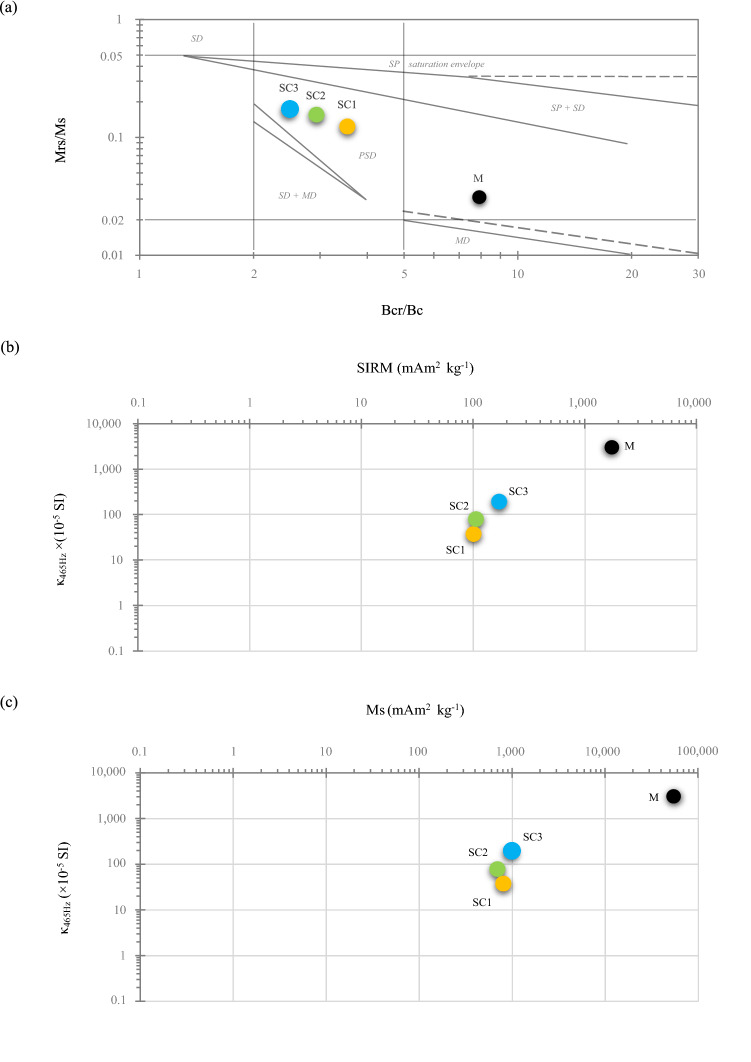
Illustration of hysteresis results for magnetite/maghemite (M) and soil concretion (SC1-3) samples. (**a**) The Day diagram^57^ with modification of Dunlop^58^. Bi-logarithmic plots of: (**b**) SIRM vs. κ_465Hz_; (**c**) Ms vs. κ_465Hz_. Bcr—coercivity of remanence; Bc—coercive force; SIRM or Mrs—remanent saturation magnetization; Ms—induced saturation magnetization; κ_465Hz_—volume magnetic susceptibility.

The dimensions of magnetic grains decrease in the following order: MD > PSD > SD > SP^42^. The distribution of the data obtained for soil concretions (SC1-3) in the Day diagram^57^, modified by Dunlop^58^, in the region of PSD grains (Fig. 3a) probably results from the co-existence of minerals with different kinds of magnetic behavior, however, with a predominance of the SFMs, i.e. magnetite/maghemite (Fig. 2b). The coercivity (Bcr/Bc) and magnetization (Mrs/Ms) ratios allow for recognition of the SC samples as PSD grains, i.e. in the region that is characteristic for the mixture of several types of domain states^58^. The position of SCs close to and above the region of single-domain and multidomain grains (SD-MD) may indicate a relatively high amount of SD grains in the studied samples (Fig. 3a). The presence of SD and not SP grains, despite the relatively high values of κ_fd_% (> 10%; Table 1), may be confirmed by the fact that the κ_fd_% parameter measured with MS2B sensor is sensitive to the presence of ferrimagnetic grains near the SP/stable SD boundary. On the other hand, the relationship between the grain distribution and the reduced blocking volume (i.e. the boundary between SP and stable SD grains) causes a decrease in the values of κ_fd_%, and can reduce κ_fd_% by over 50% for certain grain distributions^59^. Domain-state variations for two or more co-occurring magnetic minerals within a single sample may be associated with different particle size ranges for each mineral^60^. Moreover, the particles of identical size but different stoichiometry could have different domain states, and the particle sizes at which the SP to stable SD, stable SD to PSD, or PSD to MD transitions occur vary from mineral to mineral^60^. Nevertheless, the position of the magnetite/maghemite sample (M) clearly indicates the dominance of the MD grains of the primary mineral (i.e. GMPs), even despite a slight shift in the Day diagram (Fig. 3a). However, there are many variables (e.g. magnetic mineralogy, stoichiometry of minerals, or mixtures of magnetic particles), which limit the interpretation of the Day diagram in terms of the diagnosis of domain state^60^.

Generally, the relation of SIRM versus κ is used to obtain information on magnetic granulometry as well as mineralogical transformations of ferrimagnetic phases in samples, whereas, the relationship between Ms and κ is used to estimate the concentration of ferrimagnetic particles^43^. The relationships between both SIRM and Ms versus κ_465Hz_ are displayed in Fig. 3b,c. The studied samples are located diagonally, which indicates that ferrimagnetic minerals prevail, i.e. magnetic susceptibility is mainly produced by magnetite/maghemite. Moreover, in terms of the plots (SIRM versus κ_465Hz_ and Ms versus κ_465Hz_; Fig. 3b,c), it is possible to distinguish a cluster of soil concretions (SC1-3) from a magnetite/maghemite sample (M). The nearly linear distribution of data points in the bi-logarithmic plot reflects the concentration effect rather than the grain size (Fig. 3b,c). The value of Bcr should be lower for the mineral with a significant contribution of SP grains than for MD grains as the fine-grained mineral only contributes to κ and not to the SIRM or Bcr^44^ as in the case of SCs and M samples (Table 1).

## Conclusions

This experimental study brings new, very important information for the proper use of the frequency-dependent feature of magnetic susceptibility as a parameter used for the estimation of pedogenesis mostly in areas with strongly magnetic geology. Our results clearly prove that highly magnetic coarse grains are masking superparamagnetic grains of pedogenic origin. This masking can be assessed by using frequency dependent properties, sensitive to the superparamagnetic particles until a certain concentration threshold.

Magnetic susceptibility (κ_465Hz_) of pedogenic (superparamagnetic) grains is gradually masked as the contribution of coarse multidomain grains increases. The masking effect can be evaluated on the basis of a power trendline. The three experimental sets of samples show in principle similar features (R^2^), but expressed with different intensity (initial κ_465Hz_ values and contribution of multidomain grains). The initial κ_465Hz_ values of superparamagnetic materials of the experimental set increased approximately 4, 8, and 16 times, while, the initial κ_fd_% values decreased approximately 4, 9, and 27 times.

Our results provide a good basis for further research of the masking effect. More experiments should be carried out on a larger number of samples and various types of pedogenic superparamagnetic minerals, as well as on various coarse-grained primary ferrimagnetic minerals to produce a database, which may serve as a reliable and useful model for the quantification of magnetic contributions of geo-, pedo-, and technogenic origin in the soil. Moreover, the role of the magnetostatic interaction (i.e. superparamagnetic and multidomain particles dispersed and undispersed in a diamagnetic matrix) on frequency-dependent magnetic susceptibility needs to be clarified. For this purpose, subsequent magnetic analyses, detailed geochemical analyses as well as the granulometry have to be carried out.

## Supplementary Information

Supplementary Information.
