# Supplementary material for: Quantification of pedogenic particles masked by geogenic magnetic fraction
Source: Sci Rep. 2021 Jul 20;11:14800. doi: 10.1038/s41598-021-94039-1 (PMC8292341; doi:10.1038/s41598-021-94039-1)
Supplement: Supplementary file 1 — Supplementary Information. [file 41598_2021_94039_MOESM1_ESM.pdf]

Supplementary material
for the manuscript

Quantification of pedogenic particles masked by geogenic magnetic fraction

Marcin Szuszkiewicz[1,*], Hana Grison[2], Eduard Petrovský[2], Maria Magdalena Szuszkiewicz[1], Beata Gołuchowska[3], and Adam Łukasik[1]

[1]Institute of Environmental Engineering, Polish Academy of Sciences, 34 M. Skłodowskiej-Curie St., 41-819 Zabrze, Poland
[2]Institute of Geophysics, Czech Academy of Sciences, Boční II/1401, 141 00, Prague 4, Czech Republic
[3]Institute of Environmental Engineering and Biotechnology, Faculty of Natural Sciences and Technology, University of Opole, 6 Kardynała B. Kominka St., 45-032 Opole, Poland
[*]marcin.szuszkiewicz@ipis.zabrze.pl

Sample	Sampling Site Location	Coordinate	ASL *(m)*
Q	Lower Silesian Voivodeship (SW Poland)	N50.850780° E15.440964°	1
M	Upper Silesian Voivodeship (S Poland)	N54.778135° E17.635702°	316
SC1-3	Pomeranian Voivodeship (N Poland)	N50.187256° E19.065509°	1059

Table S1. Geographic location of the sampling sites (Q – quartz; M – magnetite/maghemite; SC1-3 – soil concretions). ASL – above sea level.

Specimen Code	κ_{465Hz}	κ_{4650Hz}	κ_{fd}	κ_{fd}%	Sample Composition		
					Matrix	Component 1	Component 2
	(× 10⁻⁵ SI)			*(%)*	*(g)*		
1	9.37	–	–	–	Q (2.9357)	M_{10} (0.0022)	–
2	19.48	–	–	–	Q (3.0365)	M_{20} (0.0043)	–
3	23.70	23.65	0.05	0.22	Q (2.7406)	M_{25} (0.0054)	–
4	53.70	53.49	0.21	0.40	Q (2.9919)	M_{50} (0.0117)	–
5	75.65	75.24	0.41	0.54	Q (2.8351)	M_{75} (0.0167)	–
6	97.25	96.92	0.33	0.34	Q (2.7034)	M_{100} (0.0219)	–
7	127.47	127.33	0.14	0.11	Q (2.9118)	M_{125} (0.0268)	–
8	154.42	153.94	0.48	0.31	Q (2.8733)	M_{150} (0.0321)	–
9	175.95	175.65	0.30	0.17	Q (2.6553)	M_{175} (0.0375)	–
10	206.50	205.81	0.69	0.33	Q (2.6942)	M_{200} (0.0431)	–
11	250.75	250.27	0.48	0.19	Q (2.7708)	M_{250} (0.0558)	–
12	303.87	303.00	0.87	0.29	Q (2.9313)	M_{300} (0.0641)	–
13	402.43	401.43	1.00	0.25	Q (2.6584)	M_{400} (0.0889)	–
14	496.34	495.11	1.23	0.23	Q (2.9025)	M_{500} (0.1108)	–
15	571.47	569.90	1.57	0.27	Q (2.7111)	$M_{>500}$ (0.1263)	–
16	37.65	33.61	4.04	10.70	Q (2.6438)	–	SC1 (0.2241)
17	47.02	42.75	4.27	9.10	Q (2.7659)	M_{10} (0.0022)	SC1 (0.2241)
18	58.73	54.33	4.40	7.50	Q (2.8641)	M_{20} (0.0043)	SC1 (0.2241)
19	62.95	58.55	4.40	7.00	Q (3.0062)	M_{25} (0.0054)	SC1 (0.2241)
20	94.95	89.85	5.10	5.40	Q (2.8723)	M_{50} (0.0117)	SC1 (0.2241)
21	112.30	107.60	4.70	4.20	Q (2.6416)	M_{75} (0.0167)	SC1 (0.2241)
22	136.90	132.65	4.25	3.10	Q (2.8173)	M_{100} (0.0219)	SC1 (0.2241)
23	165.12	160.66	4.46	2.70	Q (2.5325)	M_{125} (0.0268)	SC1 (0.2241)
24	195.47	191.17	4.30	2.20	Q (3.1059)	M_{150} (0.0321)	SC1 (0.2241)
25	217.80	213.60	4.20	1.90	Q (2.9426)	M_{175} (0.0375)	SC1 (0.2241)
26	242.35	238.20	4.15	1.70	Q (3.0133)	M_{200} (0.0431)	SC1 (0.2241)
27	283.80	279.25	4.55	1.60	Q (2.8369)	M_{250} (0.0558)	SC1 (0.2241)
28	346.52	343.00	3.52	1.00	Q (2.9850)	M_{300} (0.0641)	SC1 (0.2241)
29	443.08	439.50	3.58	0.80	Q (2.7341)	M_{400} (0.0889)	SC1 (0.2241)
30	540.82	538.10	2.72	0.50	Q (2.9972)	M_{500} (0.1108)	SC1 (0.2241)
31	613.72	611.25	2.47	0.40	Q (2.8546)	$M_{>500}$ (0.1263)	SC1 (0.2241)
32	79.40	71.23	8.17	10.29	Q (2.4141)	–	SC2 (0.3081)
33	89.07	80.60	8.47	9.50	Q (2.9966)	M_{10} (0.0022)	SC2 (0.3081)
34	99.88	91.50	8.38	8.40	Q (2.9855)	M_{20} (0.0043)	SC2 (0.3081)
35	102.90	95.69	7.21	7.00	Q (2.7412)	M_{25} (0.0054)	SC2 (0.3081)

36	134.80	126.31	8.49	6.30	Q (3.9017)	M_{50} (0.0117)	SC2 (0.3081)
37	154.05	145.25	8.80	5.70	Q (2.9448)	M_{75} (0.0167)	SC2 (0.3081)
38	177.75	168.69	9.06	5.10	Q (2.9175)	M_{100} (0.0219)	SC2 (0.3081)
39	208.17	198.50	9.67	4.60	Q (2.9928)	M_{125} (0.0268)	SC2 (0.3081)
40	232.92	223.36	9.56	4.10	Q (2.8733)	M_{150} (0.0321)	SC2 (0.3081)
41	256.35	247.65	8.70	3.40	Q (2.9527)	M_{175} (0.0375)	SC2 (0.3081)
42	286.90	278.30	8.60	3.00	Q (2.8455)	M_{200} (0.0431)	SC2 (0.3081)
43	330.05	320.50	9.55	2.90	Q (2.8962)	M_{250} (0.0558)	SC2 (0.3081)
44	382.07	372.50	9.57	2.50	Q (2.7448)	M_{300} (0.0641)	SC2 (0.3081)
45	483.33	475.12	8.21	1.70	Q (2.6946)	M_{400} (0.0889)	SC2 (0.3081)
46	582.37	574.81	7.56	1.30	Q (2.9193)	M_{500} (0.1108)	SC2 (0.3081)
47	650.87	643.73	7.14	1.10	Q (2.8224)	$M_{>500}$ (0.1263)	SC2 (0.3081)
48	191.62	171.93	19.69	10.28	Q (2.5722)	–	SC3 (0.2394)
49	201.96	181.50	20.46	10.10	Q (2.8824)	M_{10} (0.0022)	SC3 (0.2394)
50	212.17	192.23	19.94	9.40	Q (2.7956)	M_{20} (0.0043)	SC3 (0.2394)
51	213.59	194.15	19.44	9.10	Q (2.7513)	M_{25} (0.0054)	SC3 (0.2394)
52	244.49	223.47	21.02	8.60	Q (2.8274)	M_{50} (0.0117)	SC3 (0.2394)
53	267.74	247.12	20.62	7.70	Q (2.6937)	M_{75} (0.0167)	SC3 (0.2394)
54	288.94	269.00	19.94	6.90	Q (2.9224)	M_{100} (0.0219)	SC3 (0.2394)
55	319.66	298.24	21.42	6.70	Q (2.8412)	M_{125} (0.0268)	SC3 (0.2394)
56	346.71	325.22	21.49	6.20	Q (2.8883)	M_{150} (0.0321)	SC3 (0.2394)
57	367.74	347.16	20.58	5.60	Q (2.7791)	M_{175} (0.0375)	SC3 (0.2394)
58	399.99	378.81	21.18	5.30	Q (2.9947)	M_{200} (0.0431)	SC3 (0.2394)
59	440.34	418.35	21.99	5.00	Q (2.7924)	M_{250} (0.0558)	SC3 (0.2394)
60	493.36	470.15	23.21	4.70	Q (2.7628)	M_{300} (0.0641)	SC3 (0.2394)
61	594.62	571.50	23.12	3.90	Q (2.7884)	M_{400} (0.0889)	SC3 (0.2394)
62	698.95	675.90	23.05	3.30	Q (2.9425)	M_{500} (0.1108)	SC3 (0.2394)
63	763.76	743.15	20.61	2.70	Q (2.8226)	$M_{>500}$ (0.1263)	SC3 (0.2394)
64	-0.11	–	–	–	Q (2.9883)	–	–
65	3221.53	3214.50	7.03	0.22	Q (2.8565)	M (0.6948)	–
66	45.33	40.71	4.62	10.18	Q (2.7255)	–	SC1 (0.2327)
67	86.69	78.19	8.50	9.76	Q (2.8777)	–	SC2 (0.3214)
68	202.51	180.60	21.91	10.83	Q (2.6948)	–	SC3 (0.2481)

Table S2. Composition of the experimental sets of samples (M – magnetite/maghemite; SC1-3 – soil concretions) prepared for volume (κ_{465Hz} and κ_{4650Hz}) and frequency-dependent (κ_{fd} and κ_{fd}%) magnetic susceptibility measurements carried out by MS2 Bartington magnetic susceptibility meter with a MS2B sensor. Specimens 66, 67, and 68 – preliminary measurements described in subheading **Experimental procedures**.

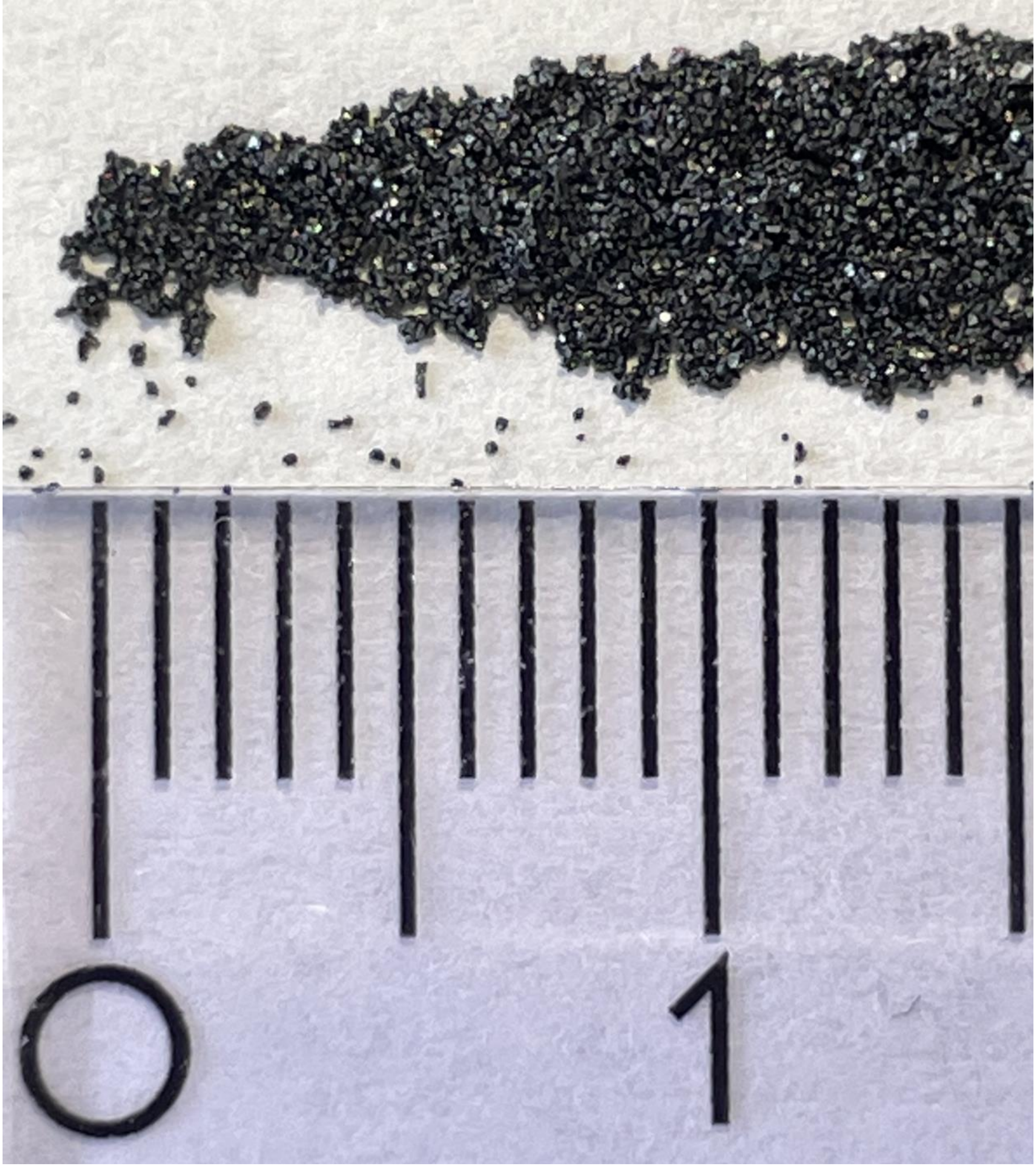

Figure S1. Photograph of a magnetite/maghemite (M) sample after the magnetic separation process (cm ruler).

(a)

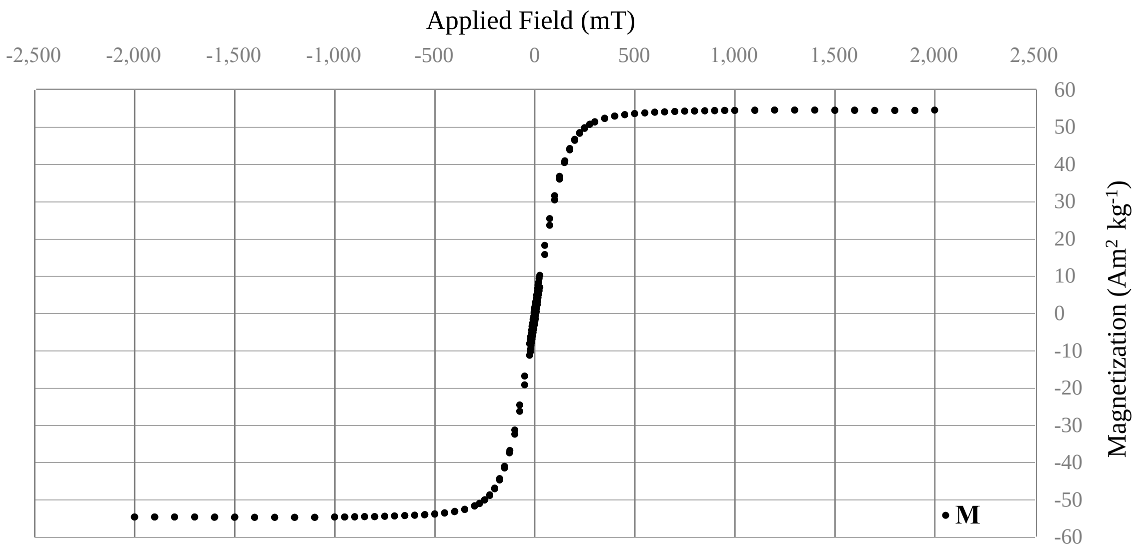

(b)

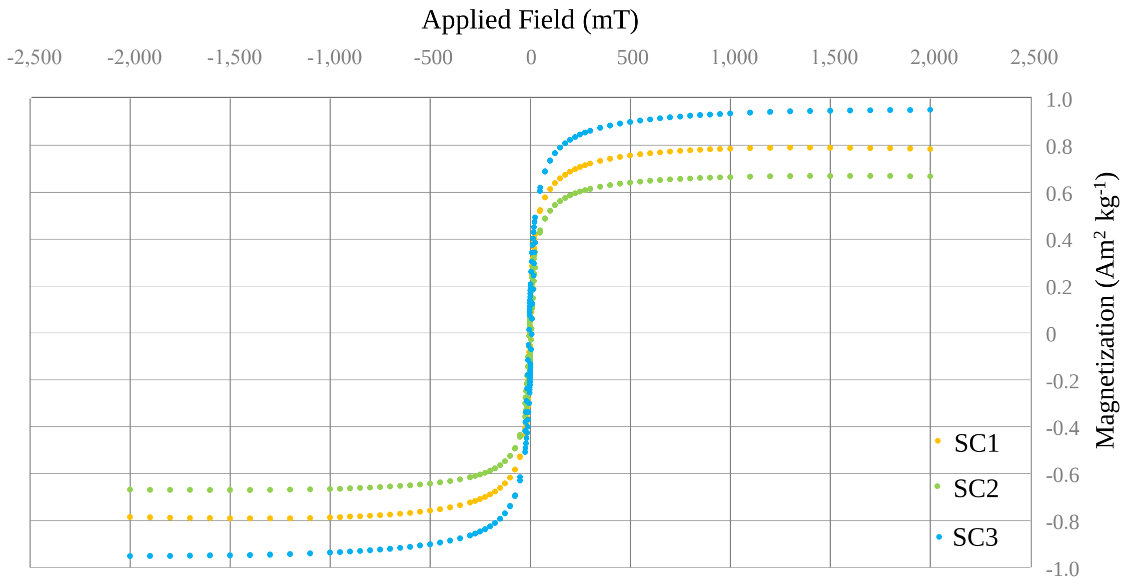

Figure S2. Hysteresis loops (after subtracting the paramagnetic contribution) of the tested samples: (a) magnetite/maghemite (M); (b) soil concretions (SC1-3).

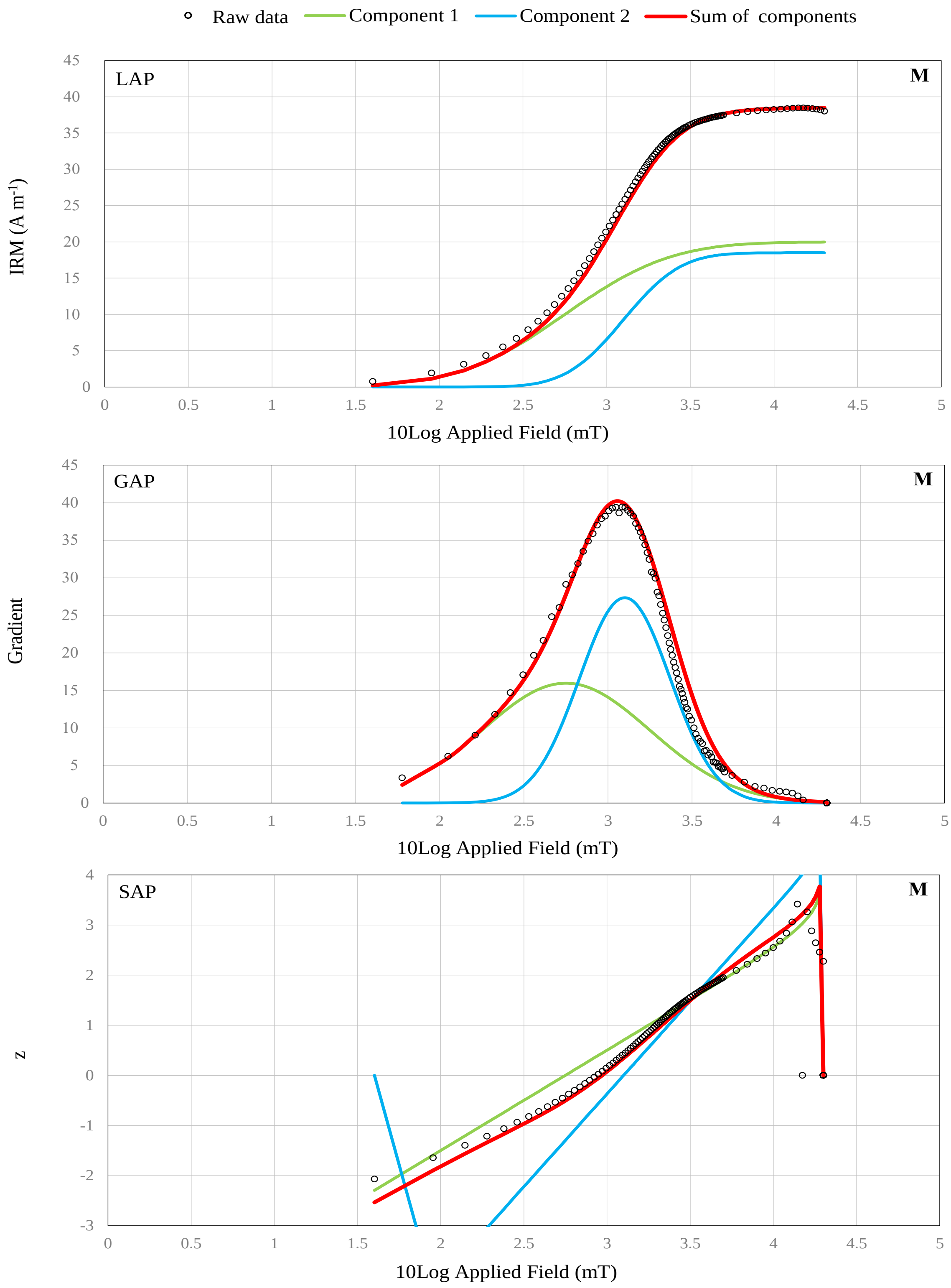

Figure S3. Linear acquisition plot — LAP, gradient of the acquisition plot — GAP and standardized acquisition plot — SAP for a real data of magnetite/maghemite (M) sample.

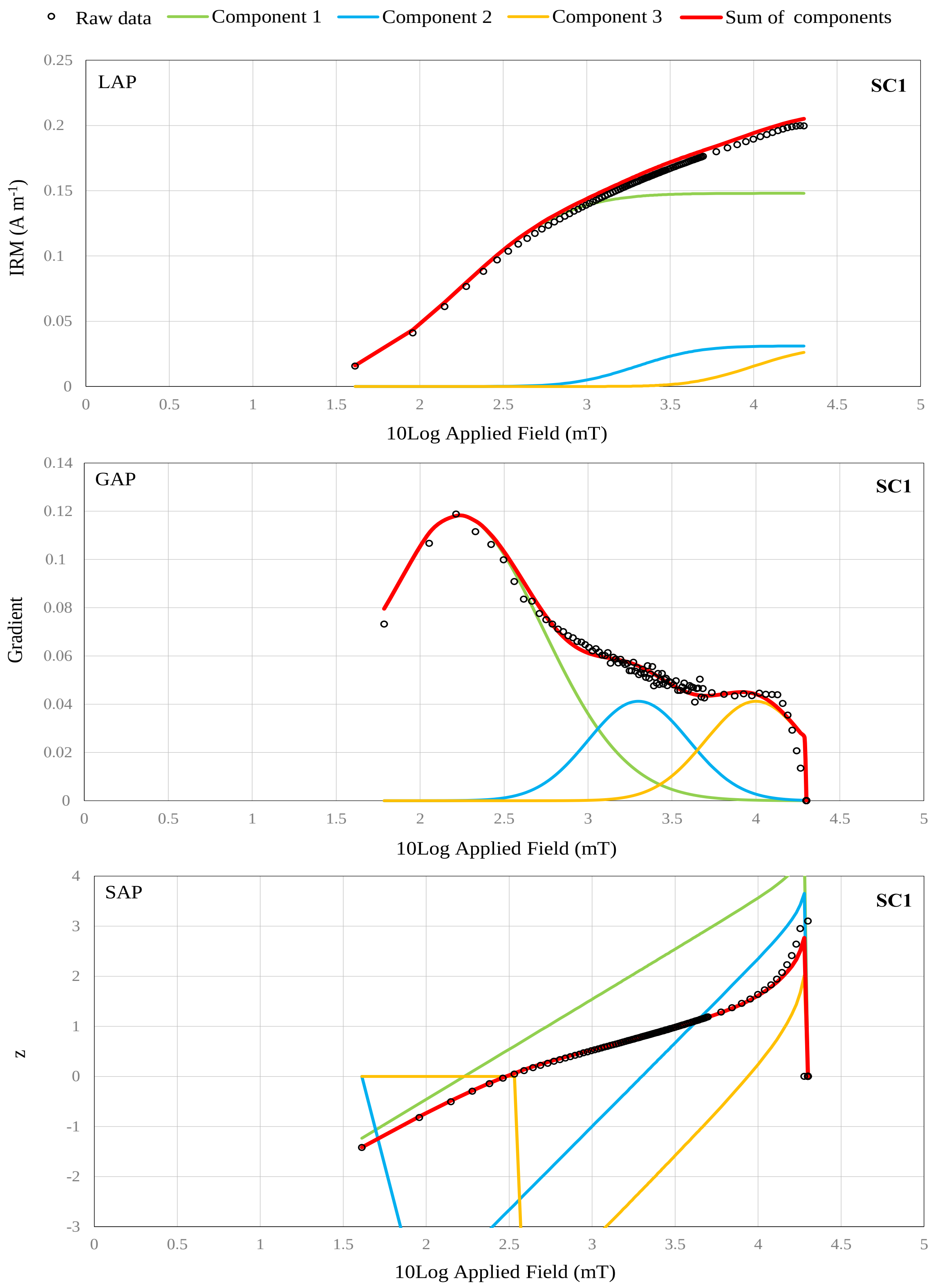

Figure S4. Linear acquisition plot — LAP, gradient of the acquisition plot — GAP and standardized acquisition plot — SAP for a real data of soil concretion (SC1) sample.

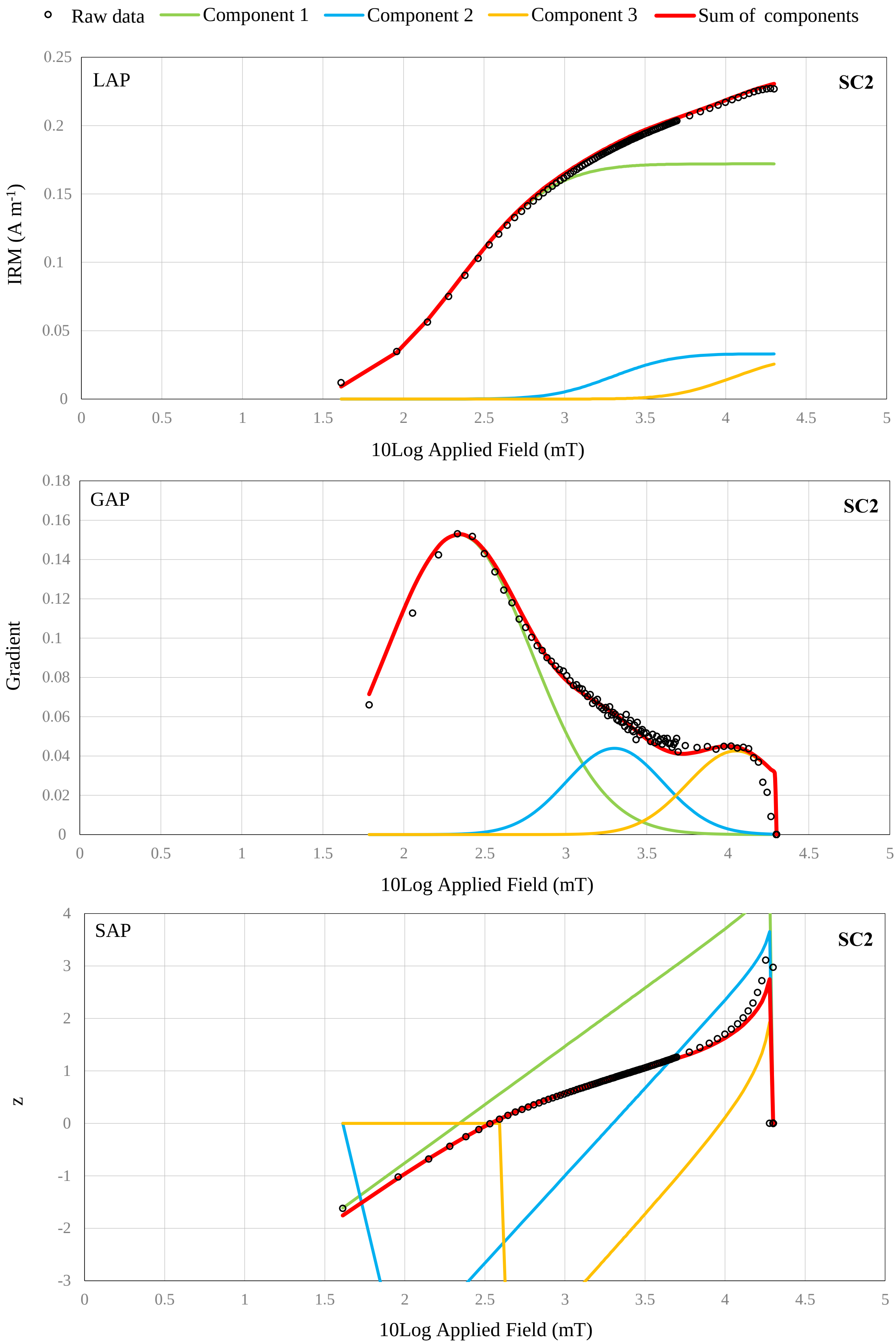

Figure S5. Linear acquisition plot — LAP, gradient of the acquisition plot — GAP and standardized acquisition plot — SAP for a real data of soil concretion (SC2) sample.

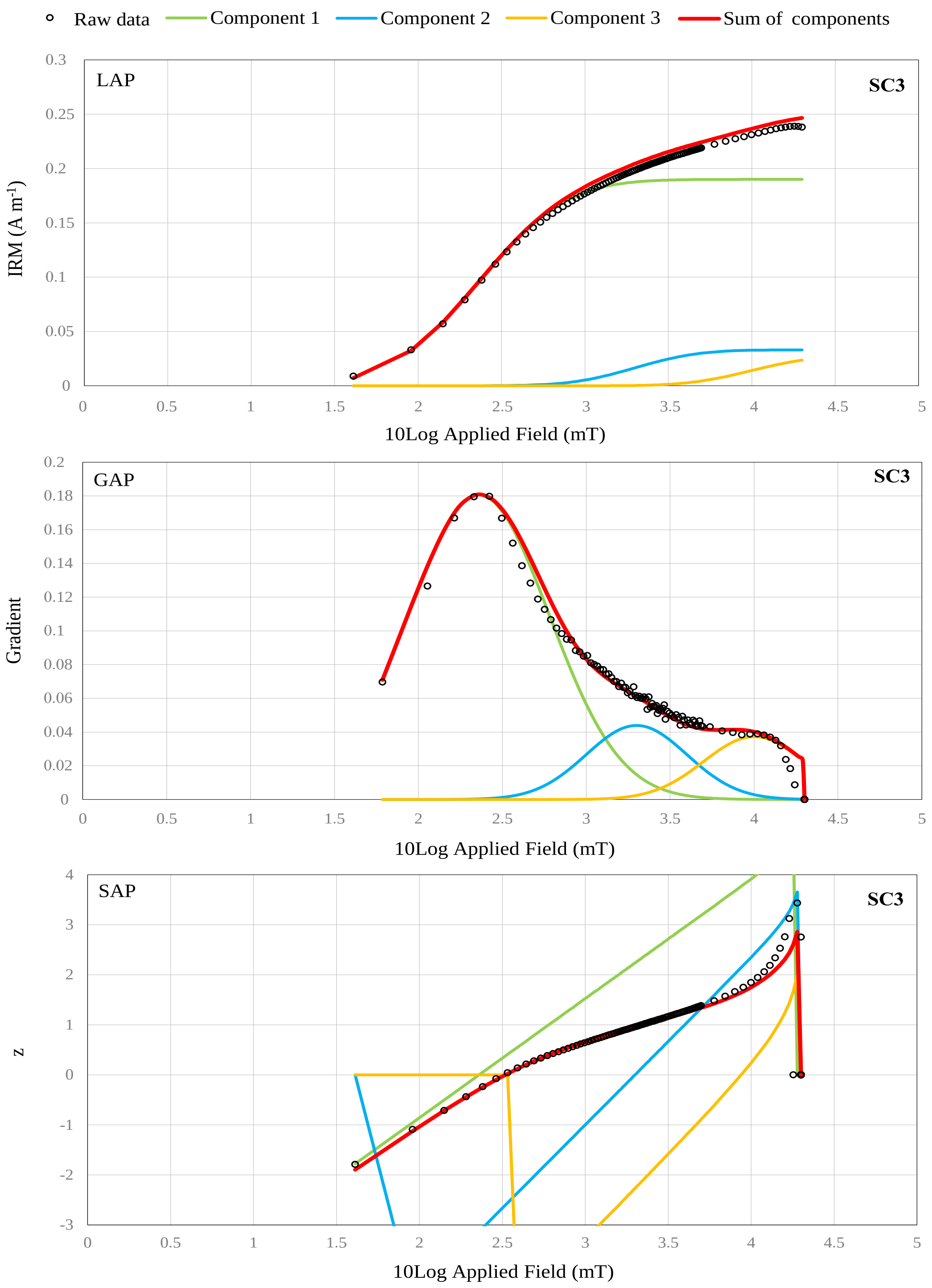

Figure S6. Linear acquisition plot — LAP, gradient of the acquisition plot — GAP and standardized acquisition plot — SAP for a real data of soil concretion (SC3) sample.

Sample	Component	Contribution	SIRM	Log($B_{1/2}$)	$B_{1/2}$	DP
		(%)	*(A m^{-1})*	*(mT)*		
M	1	51.9	20,000	2.75	56.23	0.50
	2	48.1	18,500	3.10	125.89	0.27
SC1	1	70.5	148	2.23	16.98	0.50
	2	14.8	31	3.30	199.53	0.30
	3	14.8	31	4.00	1000.00	0.30
SC2	1	72.6	172	2.34	21.88	0.45
	2	13.9	33	3.30	199.53	0.30
	3	13.5	32	4.05	1122.02	0.30
SC3	1	75.7	190	2.36	22.91	0.42
	2	13.1	33	3.30	199.53	0.30
	3	11.2	28	4.00	1000.00	0.30

Table S3. Isothermal remanent magnetization (IRM) component analyses of: magnetite/ maghemite (M) and soil concretion (SC1-3) samples. SIRM – saturation IRM; $B_{1/2}$ – field necessary to acquire half of SIRM; DP – dispersion parameter.
